# A MOORA-Based Evaluation of Printed Conductive Fabrics for E-Textile Product Design

**DOI:** 10.3390/polym18121478

**Published:** 2026-06-12

**Authors:** Elanur Demirci, Meltem Tekcin, Ismet Ege Kalkan, Esra Akgül, Elcin Emekdar-Karaman, Umut Kivanc Sahin, Simge Ozkayalar, Serhat Karakaya

**Affiliations:** 1 Department of Textile Engineering, Istanbul Technical University, Istanbul 34437, Turkey; demirciel20@itu.edu.tr (E.D.); tekcin@itu.edu.tr (M.T.); kalkani15@itu.edu.tr (I.E.K.); emekdar@itu.edu.tr (E.E.-K.); 2 Department of Industrial Design Engineering, Erciyes University, Kayseri 38030, Turkey; akgul@erciyes.edu.tr; 3 Department of Textile Engineering, Dokuz Eylul University, Izmir 35397, Turkey; simgeozkayalar@gmail.com; 4 EPS Technology, Izmir 35040, Turkey; serhat@epsteknoloji.com

**Keywords:** e-textiles, mechanical properties, decision making, multi-criteria decision-making (MCDM), Multi-Objective Optimization on the Basis of Ratio Analysis (MOORA), conductive printing

## Abstract

Electronic textiles (e-textiles) have gained significant importance due to their potential to enable wearable electronic systems. Conductive pathways in textiles can be fabricated using various approaches; among these, printing technologies stand out for their cost-effectiveness and suitability for rapid design customization. In this study, conductive patterns were produced on 100% cotton woven fabrics using rotary screen printing with different conductive paste formulations and printing layer configurations. The electrical resistance, fabric thickness, microscopic surface morphology, tensile strength, elongation, and tearing strength of the printed e-textiles were evaluated. Results indicated that resistance decreased with increasing printed track width and number of printed layers, with samples A4 and A5 exhibiting the highest conductivity. Thickness measurements and microscopic surface images showed that repeated printing increased layer build-up and surface coverage, particularly for A3 and A4. Mechanical performance tests revealed reductions in tensile strength, elongation, and tear strength after printing, attributed to restricted fiber mobility caused by the conductive paste and curing process. Despite these reductions, the mechanical property losses remained within acceptable limits for wearable applications. To determine the most suitable conductive textile for use in electronic textile product design, the Multi-Objective Optimization on the Basis of Ratio Analysis (MOORA) method, a multi-criteria decision-making (MCDM) approach, was applied using mechanical performance criteria. Electrical resistance was evaluated separately as a functional performance indicator and interpreted together with the MOORA-based mechanical ranking. Considering both mechanical and electrical performance, sample A5 was identified as the optimal alternative. Overall, this study demonstrates that printed conductive textiles can be systematically evaluated and ranked using a multi-criteria decision-making approach for material selection in wearable electronics.

## 1. Introduction

Electronic textiles (e-textiles) have attracted growing research interest because they allow sensing, signal transmission, and other electronic functions to be incorporated directly into flexible textile structures. Unlike conventional wearable devices, e-textiles can conform to the human body while preserving softness, lightness, and comfort, making them highly attractive for applications such as physiological monitoring, human–machine interaction, sports performance tracking, and healthcare-oriented Internet of Things (IoT) systems [1,2,3,4,5]. With the growing demand for comfortable and body-integrated electronic devices, the development of conductive systems compatible with textile structures has become increasingly important for the practical implementation and commercialization of wearable technologies.

Several strategies have been explored to create conductive textile structures, including weaving, knitting, embroidery, coating, lamination, and chemical finishing [2,3,6]. Among these, printing-based approaches have attracted particular attention because they enable conductive patterns to be deposited directly onto textile surfaces with high design flexibility, relatively low material consumption, and good compatibility with scalable production. Techniques such as screen printing, inkjet printing, and spray-based deposition have become widely used methods for creating conductive pathways and functional regions integrated into textile structures [5,7,8]. Compared with structural integration through conductive yarns, printing offers a more direct route for generating localized conductive tracks while maintaining the fabric architecture to a greater extent, which is especially beneficial for wearable systems requiring flexibility, breathability, and user comfort [9].

The performance of printed conductive textiles is strongly governed by both material formulation and process conditions. Silver-based inks remain among the most widely studied conductive systems because of their high electrical conductivity and suitability for printed electronics [8,10]. Silver-containing printed layers have been successfully used in flexible electronics and textile-based electrodes, demonstrating that printing can produce conductive networks with promising functional performance [7,11]. Conductive polymers, particularly poly(3,4-ethylenedioxythiophene):poly(styrene sulfonate) (PEDOT:PSS), have also attracted extensive attention because they offer a favorable combination of conductivity, flexibility, and textile compatibility. Previous studies have shown that PEDOT:PSS can be applied to cotton, polyester, and wool fabrics through printing or coating processes; however, issues such as adhesion, washing stability, and resistance to mechanical wear remain critical challenges for long-term use [12,13]. In parallel, carbon-based systems, particularly graphene-containing formulations, have become increasingly attractive as alternative conductive materials because of their flexibility and potential economic advantages. However, their performance is closely linked to dispersion stability, rheological properties, compatibility with the textile substrate, and the formation of a continuous conductive network during deposition and drying [5]. Recent studies on wearable conductive systems further indicate that the state of the art is moving beyond simple conductive pathway formation toward multifunctional materials that combine electrical performance with mechanical compliance, adhesion, durability, environmental stability, and reliable sensing capability. For example, conductive hydrogel-based wearable sensors have recently demonstrated high stretchability, stable skin adhesion, low interfacial impedance, long-term electrophysiological signal recording, anti-freezing conductivity, and machine learning-assisted gesture or attention recognition [14,15,16]. Although these hydrogel-based systems differ from printed conductive textiles in terms of material structure and fabrication route, they highlight an important design principle for wearable conductive materials: electrical performance should be evaluated together with mechanical robustness and application-relevant stability. Taken together, these studies suggest that conductive printing on textiles should be understood not simply as a deposition step but as an integrated material–process issue that ultimately shapes the electrical and functional performance of the textile.

Although conductive yarn-based textile structures remain important in the e-textile field, their performance is also known to depend strongly on structural and use-related factors. Earlier studies on knitted and woven textile electrodes demonstrated that fabric architecture, skin contact, pressure, moisture, and deformation all influence signal quality and electrical behavior [17]. Similar observations have been reported for textile sensors operating under demanding environmental conditions, where stretching, wetting, and repeated use can alter performance significantly [18]. These findings make clear that the successful design of e-textiles cannot be based on conductivity alone. For wearable applications, such as home textiles, furniture, military clothing, wearable technologies, fashion, and medical textiles, conductive structures must also exhibit sufficient mechanical integrity, durability, and application-relevant stability. This requirement is equally valid for printed conductive textiles, where the combination of conductive material, textile substrate, and printing parameters determines not only electrical resistance but also adhesion, flexibility, and resistance to mechanical damage.

Since the evaluation of conductive textile alternatives involves multiple, and often competing, criteria, it can be regarded as a multi-criteria decision-making (MCDM) problem. MCDM methods have been widely applied in engineering and material selection to enable systematic ranking in complex decision-making contexts [19,20,21,22,23,24]. In textile and related material systems, they have been applied to the selection of communicating textiles, e-textile manufacturing processes, cotton fibers, cotton fabrics, conductive yarns, and composite materials [25,26,27,28,29]. These studies show that MCDM provides an effective framework for comparing alternatives when performance cannot be represented by a single indicator.

However, existing studies on printed conductive textiles have primarily focused on conductivity improvement, sensing functionality, washability, electrochemical behavior, or durability as separate topics [5,7,8,11,12,13]. At the same time, textile-related MCDM studies have largely focused on fiber, yarn, fabric, or manufacturing process selection rather than the ranking of printed conductive textile alternatives based on experimentally measured electrical and mechanical properties [6,25,26,27,28]. Therefore, limited attention has been given to the simultaneous evaluation of electrical and mechanical performance in printed conductive textiles through a structured MCDM framework. A conductive textile with low electrical resistance cannot be regarded as practically suitable for wearable applications unless it also exhibits adequate mechanical performance.

MCDM methods are widely used to support decision problems involving multiple and often conflicting criteria, particularly when alternatives must be evaluated, compared, and ranked in a systematic manner. These approaches help decision-makers reach a trade-off solution between the best and worst options by considering finite performance criteria across different alternatives. Among the available MCDM techniques, the Multi-Objective Optimization on the Basis of Ratio Analysis (MOORA) has gained considerable attention and has been applied across a broad range of fields due to its practical structure and decision-making capability [30]. Compared to other MCDM methods, MOORA was selected because it offers advantages in terms of computation time, simplicity, reduced number of mathematical operations, and consistency [30,31].

**Figure 1:**
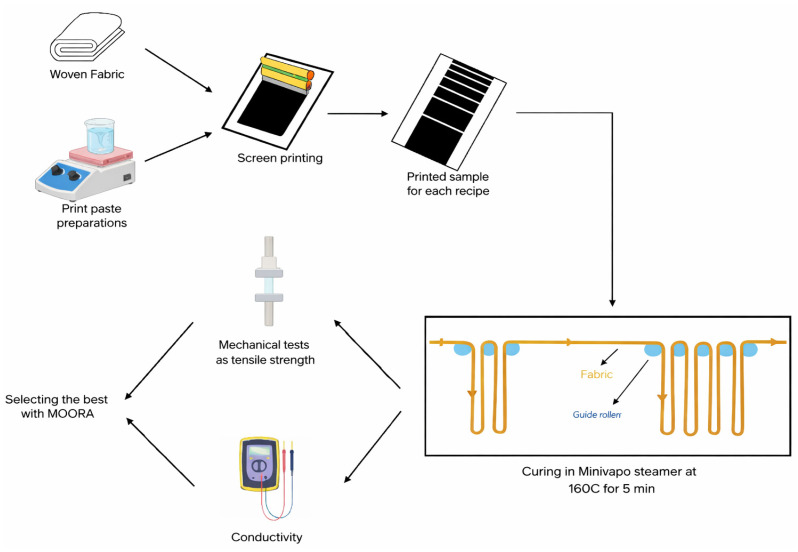
Workflow of conductive printing and electrical/mechanical characterization.

In this study, different conductive paste recipes were prepared and applied to 100% cotton woven fabric in varying widths via screen printing. After printing, mechanical and electrical tests were performed on the fabrics. The most suitable sample for e-textile applications was determined with the MOORA method, as illustrated in Figure 1. Accordingly, this study aimed to evaluate printed conductive textile alternatives by considering both electrical and mechanical performance and to identify the most suitable option using the MOORA method as a multi-criteria decision-making approach. The printing strategies used to create conductive pathways were found to influence both the electrical resistance and the mechanical performance of the fabrics. However, electrical resistance was not directly included in the MOORA decision matrix because the resistance values were strongly dependent on measurement geometry, including probe distance and printed track width. Therefore, a single electrical resistance value would not fully represent the overall electrical behavior of each sample and could introduce bias into the multi-criteria ranking. For this reason, the MOORA analysis was focused on mechanical performance criteria, namely tensile strength, breaking elongation, and tearing strength, while electrical resistance was evaluated separately as a functional performance indicator. The final interpretation was made by considering both the MOORA-based mechanical ranking and the electrical resistance results. The study contributes to the literature in three main respects. First, it provides a comparative evaluation of conductive printed textile samples produced under different material and process conditions. Second, it applies MOORA as a systematic quantitative tool for ranking conductive textile alternatives on the basis of multiple performance criteria. Third, it proposes a practical decision-making framework that may support material and process selection in wearable e-textile design.

## 2. Materials and Methods

### 2.1. Materials

In this study, 100% cotton woven fabrics were employed. The fabric had an areal density of 238 g/m^2^ and a satin weave structure with a warp yarn count of Ne 30 and a weft yarn count of Ne 10. The fabric density was 55 ends and 55 picks per cm. The fabrics with the given properties were printed using conductive printing pastes on a laboratory-type rotary printing machine. The properties of the conductive paste applied onto the fabric surfaces are given in Table 1.

The paste formulations were selected to compare different routes for obtaining conductive printed surfaces on cotton woven fabrics. A1 was prepared as a simple acrylic copolymer-based carbon black formulation, while A2 represented a conventional pigment printing paste containing the same amount of carbon black together with standard textile printing auxiliaries. This comparison was intended to show the effect of the paste system on the final electrical and mechanical performance. A3 and A4 were prepared with a lower carbon black content but with repeated printing layers in order to evaluate whether layer build-up could improve conductive network formation. Tubicoat ECH was included as a commercial, ready-to-use carbon-based conductive coating paste and used as a reference system for textile-oriented conductive coatings. Since this paste is already formulated for conductive textile coating applications, it provided a practical benchmark for comparing the laboratory-prepared paste recipes with a commercially available product. This was particularly useful for evaluating whether a single-layer commercial paste could provide comparable or improved conductivity and mechanical performance when compared with the laboratory formulations. Therefore, the selected formulations were not arbitrary but were chosen to compare the effects of paste composition, conductive material loading, repeated layer deposition, and commercial conductive paste performance on the electrical and mechanical behavior of the printed fabrics.

### 2.2. Methods

#### 2.2.1. Printing Process

**Figure 2:**
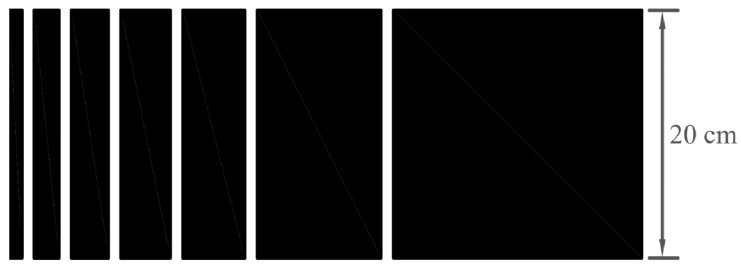
Image of the printed pattern.

Before printing, the desired pattern was designed and transferred onto a stencil with dimensions of 150 cm in width and 64 cm in length. The prepared conductive printing pastes were then applied to fabrics. An image of the printed pattern is presented in Figure 2. As shown in the figure, stripe patterns with a constant length of 20 cm and varying widths of 1, 2, 3, 4, 5, 10, and 20 cm were printed. In addition, a full-background printed area with a length of 20 cm was also produced. This design enabled the investigation of the electrical conductivity behavior of printed stripes with different widths. Repeated printing was also carried out on the fabric surfaces in order to compare the electrical resistance values obtained under different printing conditions.

The fabrics were printed using a laboratory-scale rotary printing machine (Mini MDK, J. Zimmer, Maschinenbau GmbH, Klagenfurt, Austria) with a 135-mesh screen; a squeegee diameter of 12 mm; a squeegee pressure setting of 5, corresponding to 50 N; and a printing speed of 20 m/min, according to the formulations given in Table 1. These printing parameters were kept constant for all samples in order to ensure comparability among the different paste formulations. After each printing layer, including the repeated layers in A3 and A4, the printed fabric was dried at 110 °C for 2 min. Subsequently, the samples were fixed at 160 °C for 5 min in a curing machine (MiniVapo 80, Arioli, Italy). For multilayer samples, the same drying and fixation sequence was applied after each printed layer before the next layer was deposited.

#### 2.2.2. Mechanical Properties of Printed e-Textiles

The mechanical performance of the printed e-textiles was evaluated in terms of tensile strength, elongation, and tearing strength using a tensile testing machine (QC508M1F, Cometech, Taichung City, Taiwan).

Tensile strength and elongation tests were determined in accordance with ISO 13934-1 [32], using the strip method. For these tests, 5 specimens were prepared for each sample with an initial width of 60 mm and a length of 300 mm, including the clamping allowance. Threads were removed from both sides of each specimen to form fringed edges, and the final test width was adjusted to 50 mm. For each sample, five specimens were tested in both the warp and weft directions, and the mean tensile strength and breaking elongation values were reported.

Tearing strength was determined according to ISO 13937-2 [33] using the trouser tear method. In this method, five rectangular specimens from each sample with dimensions of 200 × 50 mm were prepared, and a central cut was introduced on the short side to form a trouser-shaped test specimen. During the test, each leg of the specimen was clamped separately, with the notch positioned at the midpoint between the jaws, and the mean tearing force value of five repeats was recorded.

Because the tensile and tear tests were performed in accordance with the relevant textile testing standards, both specimen preparation and test procedures followed the conditions defined by these standards. The resulting mechanical data were subsequently used as performance criteria in the multi-criteria evaluation of the printed conductive textile alternatives.

#### 2.2.3. Electrical Properties of Printed e-Textiles

**Figure 3:**
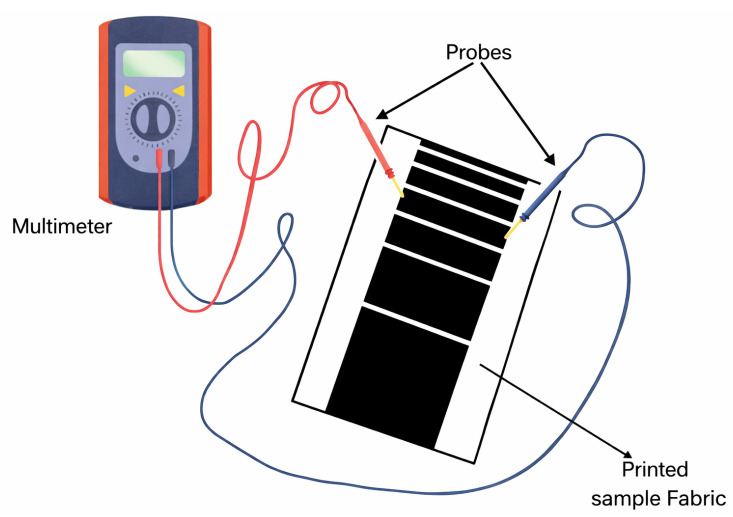
Measurement of electrical resistance.

The electrical performance of the printed e-textiles was assessed through surface resistance measurements, which were used as an indicator of electrical conductivity. Surface resistance values were determined using the two-probe method because of its practical applicability and short measurement time. All measurements were performed using a UNI-T PRO digital multimeter (Uni-Trend Technology (China) Co., Ltd., Dongguan, China), as shown in Figure 3.

For each fabric sample, measurements were taken from ten different locations on the printed surface. At each location, 5 repeated measurements were performed, and the average resistance value was calculated. To evaluate the effect of conductive path length on electrical resistance, the distance between the two probes was systematically varied. Accordingly, resistance values were measured at probe spacings of 1, 2, 3, 4, 5, 10, 15, and 20 cm along the printed fabric surface.

In addition, stripe patterns with different widths (1, 2, 3, 4, 5, 10, and 20 cm) and a constant length of 20 cm were printed in order to examine the influence of printed width on electrical conductivity. The electrical resistance values obtained from these measurements were then used to compare the conductive performance of the printed samples.

#### 2.2.4. Thickness Measurement of Printed Fabrics

The thickness of the unprinted and printed fabrics was measured in accordance with ASTM D1777 [34] using a James H. Heal R&B cloth thickness tester (James Heal, Halifax, UK). Since the samples were woven fabrics, the measurements were performed under an applied pressure of 4.14 ± 0.21 kPa, as specified in the standard. For each laboratory sampling unit, ten thickness measurements were taken from different locations on the fabric surface, and the average thickness value was reported. Measurements were performed to evaluate the change in fabric thickness after conductive printing and to estimate the apparent thickness contribution of the printed conductive layer. Since the measurements were carried out on textile substrates, the reported values represent the total fabric thickness after printing rather than the isolated thickness of the conductive layer. The apparent thickness increase was calculated by subtracting the thickness of the unprinted fabric from that of each printed sample.

#### 2.2.5. Microscopic Surface Examination

Microscopic surface examination was performed to evaluate the surface morphology, coating uniformity, and local paste accumulation of the unprinted and printed fabric samples. The images were obtained using a Motic BA300 microscope (Motic, Xiamen, China) at 10× magnification. The microscopic images were used to support the interpretation of electrical resistance results in relation to fabric surface roughness, printing uniformity, and conductive layer continuity.

#### 2.2.6. The Multi-Objective Optimization on the Basis of Ratio Analysis (MOORA)—Method

In the MOORA analysis, the alternatives were the five printed conductive fabric samples, namely A1–A5. Six mechanical performance criteria were considered: tensile strength in the weft direction, tensile strength in the warp direction, breaking elongation in the weft direction, breaking elongation in the warp direction, tearing strength in the weft direction, and tearing strength in the warp direction. Since higher values of these criteria indicate better mechanical performance, all criteria were treated as beneficial criteria.

The steps of the MOORA method for this study are as follows:

Step 1. Construction of a decision matrix

In the MOORA method, the first step involves constructing the decision matrix, as shown in Equation (1) [35,36]. In the ratio system, each value of an alternative is compared with a denominator representing all alternatives associated with a specific attribute or objective.

(1) $$X=\left[\begin{array}{c} \chi_{11}\;\chi_{12}\;\ldots \;\chi_{1n}\\ \chi_{21}\;\chi_{22}\;\ldots \;\chi_{2n}\\ \chi_{31}\;\chi_{32}\;\ldots \;\chi_{3n}\\ \ldots \;\ldots \;\ldots \;\ldots \\ \chi_{m1}\;\chi_{m2}\ldots \;\chi_{mn} \end{array}\right]$$

where xij performance measure of i-thalternative on j-th attribute, m is the number of alternatives, and n is the number of attributes.

Step 2. Normalization of data

For the normalization process, the denominator is defined as the square root of the sum of squares of all alternatives with respect to each attribute or objective. After normalization, the scores or responses of alternatives concerning the objectives become dimensionless, falling within the range of [0, 1] or [−1, 1], depending on the problem context. The data on conductive fabrics’ mechanical properties are normalized with Equation (2). The objective of normalization is to ensure the consistency of relationships between data.

(2) $$X_{ij}^{*}=\frac{\chi_{ij}}{\sqrt{\sum_{i=1}^{m}\chi_{ij}^{2}}}\;i\;=\;1,\;2,\ldots \ldots m;\;j\;=\;1,\;2,\ldots \ldots r$$

Step 3. The ratio system

The normalized values are aggregated for beneficial objectives and subtracted for non-beneficial objectives, and the algebraic sum of these two components yields the normalized assessment values of the alternatives, which serve as the basis for ranking. The MOORA index is calculated using Equation (3), in which the normalized values are added in the case of maximization and subtracted in the case of minimization for each alternative [35,36].

(3) $$Y_{ij}^{*}=\sum_{j=1}^{t}Y_{ij}-\sum_{j=t+1}^{r}Y_{ij}$$

where t is the number of maximized responses, (r − t + 1) is the number of responses to be minimized, and ($Y_{ij}^{*}$) is the ranking score of scenarios with respect to all responses.

Step 4. Ranking alternatives

According to Brauers and Zavadskas [35], this method, which derives the final performance of candidate alternatives based on the ordered ranking of assessment values, is highly robust in terms of decision-makers (stakeholders), criteria (objectives), and the interrelationships between criteria and alternatives. According to the obtained MOORA score, the results show that the highest ($Y_{ij}^{*}$) value is the best alternative, while the worst alternative has the lowest ($Y_{ij}^{*}$) value.

In the present study, the MOORA method was applied using mechanical performance criteria only, including tensile strength, breaking elongation, and tearing strength. Since higher values for these parameters indicate improved mechanical integrity, all criteria were treated as beneficial. The resulting ranking therefore reflects the relative mechanical performance of the printed conductive textile samples. Electrical resistance was not included as a direct criterion in the MOORA decision matrix because the measured resistance values were not single-point material constants but depended on both probe spacing and printed track width. Since the electrical measurements were obtained over a matrix of different distances and widths, selecting only one resistance value as a MOORA criterion could have biased the ranking toward a specific geometry. Therefore, the MOORA model was constructed using mechanical performance criteria only, which were directly comparable among all alternatives. Electrical resistance was evaluated separately and used as a functional verification parameter when interpreting the final suitability of the printed conductive textile samples. The MOORA calculations, bar graphs, and heatmap visualizations were prepared using Microsoft Excel 2024 (Microsoft Corporation, Redmond, WA, USA).

## 3. Results

### 3.1. Investigation of Electrical Conductivity Properties of Printed e-Textiles

**Figure 4:**
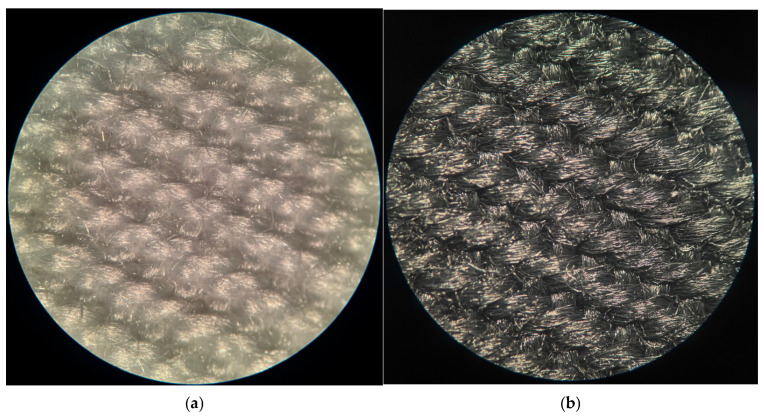

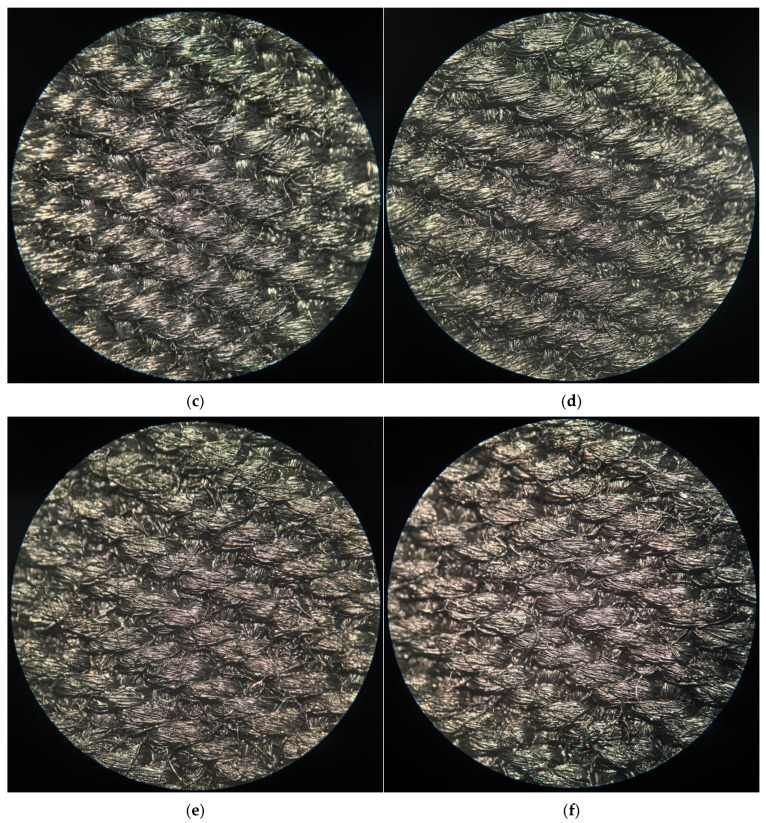
Microscopic surface images of the unprinted and printed fabric samples: (**a**) without printing, (**b**) A1, (**c**) A2, (**d**) A3, (**e**) A4, and (**f**) A5. The images show the surface morphology, printing uniformity, and distribution of the conductive layer on the fabric surface.

Microscopic surface images were examined to support the interpretation of the electrical resistance results, as shown in Figure 4. The unprinted fabric showed the typical white surface of the woven cotton substrate. After printing, A1 and A2 exhibited black surfaces with relatively thin and uniform coverage, and no pronounced paste accumulation was observed. In A3, the second printing layer produced a darker and denser surface, with local conductive paste accumulations in some regions. This effect was more pronounced in A4, where the three-layered structure resulted in a thick and dense coating layer on the fabric surface. Such layer build-up may have promoted the formation of more continuous conductive pathways, contributing to the lower resistance values observed for A4. Although A5 was produced with a single printing layer, it showed better surface coverage than A1 and A2, indicating the effective coating ability of the commercial polyurethane and carbon-based conductive paste. Overall, the microscopic images demonstrate that surface roughness, coating uniformity, local paste accumulation, and the number of printed layers affect conductive pathway formation and may therefore contribute to variations in electrical resistance.

**Figure 5:**
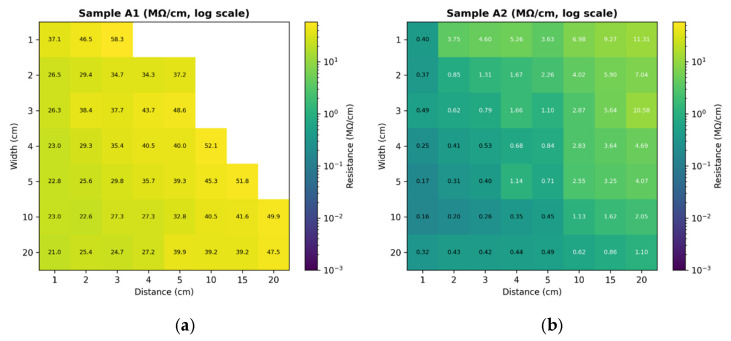

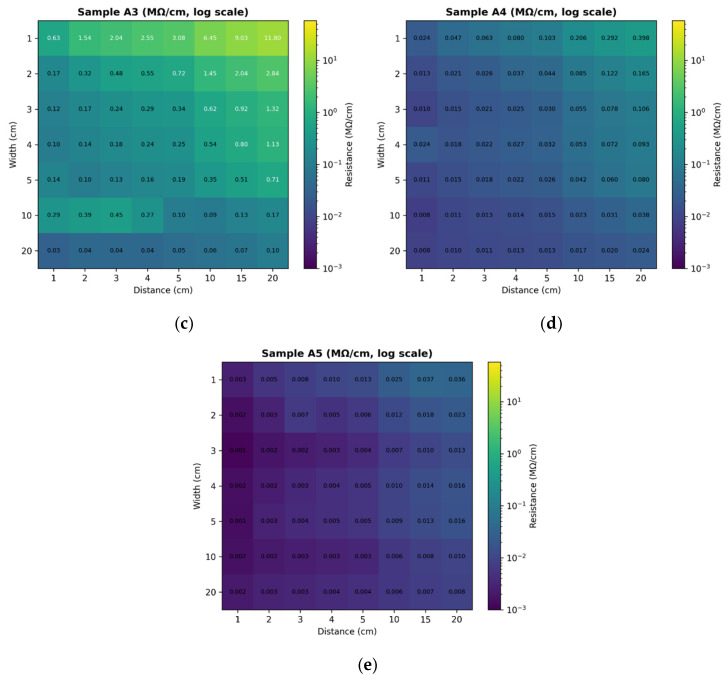
Heatmap representation of the electrical resistivity of the printed conductive textile samples as a function of probe distance and printing width: (**a**) A1, (**b**) A2, (**c**) A3, (**d**) A4, and (**e**) A5.

The electrical resistance values of the electronic textiles were measured, and the electrical resistance measurement results are given in Figure 5. A heatmap representation was used to visualize the relationship between probe distance and printing width on electrical resistance. The results clearly indicate that resistance increases with increasing probe distance, while wider printed tracks tend to exhibit lower resistance values, as expected.

The electrical resistance results indicate that resistance generally increased with increasing probe spacing, whereas resistance per unit length tended to decrease as the printed track width increased. This trend highlights the strong influence of print geometry on the electrical behavior of all samples. In general, thicker stripe patterns showed improved conductivity; however, some deviations from this tendency were observed, which can be attributed to printing irregularities and the inherent roughness of the fabric surface.

Among all samples, A5 and A4 exhibited the lowest resistance values, indicating the highest electrical performance within the group. A2 and A3 showed intermediate resistance levels and therefore lower conductivity compared with A4 and A5. In contrast, A1 consistently exhibited the highest resistance values over the investigated probe distances and print widths. For A1, no measurable resistance could be obtained at larger probe distances under low print-width conditions, whereas such a limitation was not observed for A2, A3, A4, or A5. This behavior suggests that the inferior electrical performance of A1 may be associated with differences in printing paste composition.

When the results are evaluated in relation to the number of print layers, repeated printing appears to improve the electrical conductivity of the fabrics. This effect is clearly observed in the comparison between samples A3 and A4, both printed on 100% cotton fabric, where the three-layer printed sample (A4) exhibited lower resistance values than the two-layer printed sample (A3). In addition, the woven fabric printed with the commercially available conductive paste (A5) showed the lowest resistance values overall, as expected, and therefore the highest conductivity performance among all samples.

Electrical conductivity requirements in conductive textiles are strongly application-dependent. The literature indicates that relatively modest conductivity may be sufficient for simpler functions such as antistatic behavior, whereas more demanding uses, including electromagnetic shielding, signal transmission, and wearable electronic textiles, require higher conductivity and correspondingly lower electrical resistance [37]. From this perspective, the electrical performance obtained in the present study should be evaluated according to the intended application, and samples exhibiting lower resistivity can be regarded as more promising candidates for advanced conductive textile applications.

### 3.2. Thickness of Printed Conductive Fabrics

The thickness values of the unprinted and printed fabric samples are presented in Table 2. The unprinted fabric had a thickness of 503.0 µm. After printing, all samples exhibited an increase in total fabric thickness, although the magnitude of this increase depended on the paste composition and the number of printed layers. A1, A2, and A5 showed only slight increases in thickness, whereas A3 and especially A4 exhibited more pronounced thickness increases due to repeated printing. The highest thickness was measured for A4, which was printed with three layers, indicating that layer build-up directly contributed to the increase in fabric thickness.

### 3.3. Investigation of the Mechanical Properties of Printed e-Textiles

**Figure 6:**
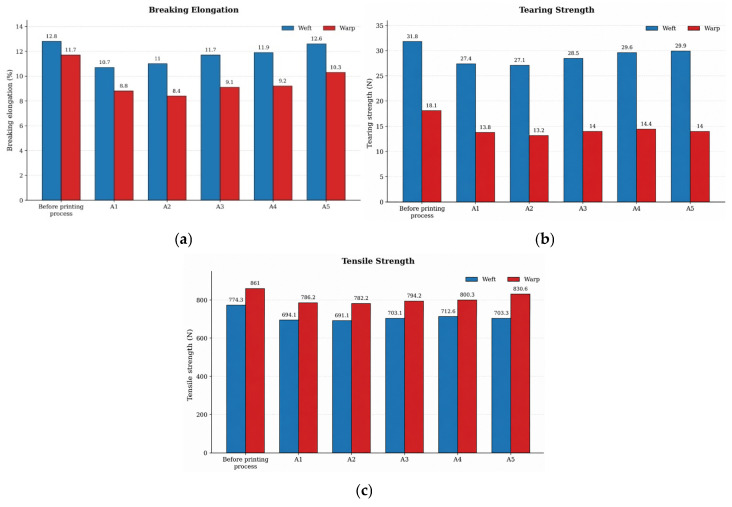
Mechanical Performance of the printed conductive textile samples: (**a**) breaking elongation, (**b**) tearing strength, and (**c**) tensile strength.

Strength is one of the most important properties that determines the lifespan of textile materials. Furthermore, conductive textiles should maintain the properties of conventional textile structures in addition to their electrical properties. Therefore, the tensile strength, breaking elongation, and tearing strength of the conductive printed samples were measured using a tensile strength tester. The physical test results of the samples are shown in Figure 6.

The mechanical performance of the fabrics was clearly affected by the conductive printing process. After printing, all samples exhibited reductions in tensile strength, breaking elongation, and tearing strength due to the formation of a rigid coating layer and reduced fiber mobility [38,39]. Compared with the unprinted fabrics, tensile strength decreased by approximately 8–12% in both the warp and weft directions for samples A1–A5. This reduction is likely associated with partial stiffening of the fabric structure during printing and curing, which may have limited fiber mobility. A similar downward trend was observed for breaking elongation, with the reduction being more pronounced in the warp direction. Tearing strength also declined in all samples, which may be attributed to the restriction imposed by the printed conductive layer on yarn and fiber movement, thereby limiting the deformation mechanisms that normally contribute to tear resistance, as also reported for coated and functionalized textile structures [38].

Despite these reductions, the extent of mechanical deterioration varied among the samples. In particular, A4 and A5 retained higher tensile strength values after printing than the other groups, suggesting that their structural integrity was less affected by the printing process. Overall, although conductive printing led to some loss in mechanical performance, the reductions remained within an acceptable range for conductive textile applications, since such systems are expected to preserve sufficient mechanical integrity to withstand bending, stretching, and handling during use [3].

Prior to the MOORA analysis, the experimental mechanical data were normalized to remove scale differences among the criteria and to ensure a comparable evaluation of all samples. This step was necessary because tensile strength, breaking elongation, and tearing strength were measured in different ranges and could otherwise affect the ranking unevenly. The normalized values obtained using the standard MOORA normalization procedure are given in Table 3.

Finally, the ranking of all alternatives specified in step 4 was determined according to the MOORA ratio method, and the rankings are given in Table 4.

According to the MOORA ratio results, the conductive fabric samples can be ranked in descending order as A5 > A4 > A3 > A1 > A2 based on their mechanical performance. Among the evaluated alternatives, A5 exhibited the highest overall performance, while A2 showed the lowest score. These findings indicate that the mechanical response of the samples was not affected to the same extent by the printing process. When interpreted together with the electrical resistance measurements, the ranking further supports the selection of A5 as the most suitable conductive fabric alternative among the tested samples.

## 4. Discussion

This study evaluated the electrical and mechanical performance of conductive patterns printed on 100% cotton woven fabrics and used the MOORA method to identify the most suitable conductive textile alternative for wearable electronic applications. The results showed that both print geometry and the number of printing layers had a substantial effect on electrical behavior. Wider printed tracks and repeated printing reduced electrical resistance, and samples A4 and A5 exhibited the best conductivity performance among the tested fabrics.

Conductive printing also led to slight reductions in tensile strength, breaking elongation, and tearing strength in all samples. These changes were mainly associated with the stiffening effect of the printed conductive layer and the corresponding restriction of fiber mobility. Nevertheless, the observed reductions remained within acceptable limits for wearable textile applications, and samples A4 and A5 preserved their mechanical performance more effectively than the other alternatives.

The thickness results also help explain the differences in electrical and mechanical behavior among the printed samples. In general, increasing the number of printing layers increased the total fabric thickness, as observed for A3 and A4. The higher apparent thickness of A4 may have contributed to the formation of a more continuous conductive network, which is consistent with its lower electrical resistance compared with A3. However, increased layer build-up may also increase fabric stiffness and restrict yarn and fiber mobility, thereby affecting mechanical performance. In contrast, A5 showed only a slight increase in thickness compared with the unprinted fabric, while still providing the highest conductivity performance. This suggests that the commercial polyurethane- and carbon-based conductive paste formed an efficient conductive layer without requiring a large increase in fabric thickness. Therefore, the thickness measurements support the interpretation that both conductive layer build-up and paste formulation influence the balance between electrical resistance and mechanical performance.

The MOORA-based assessment, carried out using mechanical performance criteria, ranked the samples as A5 > A4 > A3 > A1 > A2. Electrical resistance was not incorporated directly into the MOORA decision matrix because the measured resistance values were strongly dependent on measurement geometry, particularly probe distance and printed track width. Therefore, reducing the electrical response of each sample to a single resistance value could have introduced bias into the optimization process. Instead, electrical resistance was evaluated separately as a functional performance indicator and was considered together with the MOORA-based mechanical ranking in the final assessment of sample suitability. Among the evaluated alternatives, A5 was identified as the most suitable sample. This result is further supported by the electrical resistance measurements, which showed that A5 also provided the highest conductivity performance. Particularly, A5 achieved this performance without the use of additional conductive material in its formulation, simplifying the preparation process and reducing time, labor, and material consumption. Furthermore, compared to other conductive samples such as A3 and A4, A5 exhibited a more efficient conductive behavior by reaching relatively high conductivity within a single-layer structure, highlighting its potential as a more sustainable and cost-effective alternative. Taken together, these findings suggest that A5 offered the most favorable overall balance between electrical functionality and mechanical integrity.

## 5. Conclusions

In conclusion, this study shows that printing-based conductive textile production can provide technically promising results for e-textile applications when both electrical and mechanical performance are considered together. Future studies should further examine the long-term applicability of such materials by including washing durability, flexural fatigue, and conductivity stability under repeated use conditions. In addition, further research may investigate the preparation of color-responsive dyeing, printing, and coating formulations, as well as the integration of these functional surfaces with signal-transmitting e-textile structures produced from different conductive yarns. From this perspective, composite or sandwich textile architectures combined through techniques such as ultrasonic bonding, pressing, and coating may provide a promising design route. Ultimately, these approaches may support the development of reliable, durable, visually uniform, flexible, and low-energy-consumption functional e-textile structures for potential use in home textiles, furniture, military clothing, wearable technologies, fashion, and medical textile applications.

## Figures and Tables

**Table 1:** Printing paste contents applied to fabrics.

Sample Code	Paste Content	Conductive Material and Amount	Number of Printing Layers
A1	Acrylic copolymer dispersion (Helizarin ultra-fast PA)	200 g/Bercolin Black FB	1 layer
A2	Pigment printing paste (Binder, thickener, fixer, crosslinker, anti-foaming, ammonia, urea, water)	200 g/Bercolin Black FB	1 layer
A3	Pigment printing paste	100 g/Bercolin Black FB	2 layers
A4	Pigment printing paste	100 g/Bercolin Black FB	3 layers
A5	Polyurethane and carbon-based ready-made conductive paste (Tubicoat ECH)	-	1 layer

**Table 2:** Thickness values of the unprinted and printed fabric samples.

Sample	Total Fabric Thickness (µm)	Apparent Thickness Increase (µm)	Thickness Increase (%)
Without printing	503.0	-	-
A1	506.9	3.9	0.78
A2	507.2	4.2	0.84
A3	514.1	11.1	2.21
A4	526.6	23.6	4.69
A5	507.8	4.8	0.95

**Table 3:** Normalized decision matrix for the mechanical performance criteria of conductive fabric samples.

Sample	Tensile Strength		Breaking Elongation		Tearing Strength	
	Weft	Warp	Weft	Warp	Weft	Warp
A1	0.442887	0.440113	0.412531	0.428611	0.429618	0.444459
A2	0.440972	0.437874	0.424098	0.409129	0.424914	0.425135
A3	0.448629	0.444592	0.451086	0.443223	0.446865	0.4509
A4	0.454691	0.448006	0.458797	0.448093	0.464112	0.463783
A5	0.448757	0.464968	0.485785	0.50167	0.468816	0.4509

**Table 4:** The Multi-Objective Optimization on the Basis of Ratio Analysis (MOORA) ratio results of conductive fabrics.

Sample	MOORA Ratio Method ($Y_{ij}^{*}$)	Rank
A2	2.562121	5
A1	2.598218	4
A3	2.685294	3
A4	2.737483	2
A5	2.820896	1

## Data Availability

The original contributions in this study are included in the article. Further inquiries can be directed to the author.

## Abbreviations

MCDM	Multi-Criteria Decision-Making
MOORA	Multi-Objective Optimization on the Basis of Ratio Analysis
IoT	Internet of Things
PEDOT:PSS	Poly(3,4-ethylenedioxythiophene):poly(styrene sulfonate)
